# Anti-osteoclastogenic activity of matairesinol via suppression of p38/ERK-NFATc1 signaling axis

**DOI:** 10.1186/1472-6882-14-35

**Published:** 2014-01-21

**Authors:** Sik-Won Choi, Kie-In Park, Jeong-Tae Yeon, Byung Jun Ryu, Kwang-Jin Kim, Seong Hwan Kim

**Affiliations:** 1 Laboratory of Translational Therapeutics, Pharmacology Research Center, Bio-Organic Science Division, Korea Research Institute of Chemical Technology, P.O. Box 107, Yuseong-gu, Daejeon 305-600, Korea; 2 Division of Biological Sciences, College of Natural Science, Chonbuk National University, Jeonbuk 561-756, Korea; 3 Department of Pharmacy, Sunchon National University, Suncheon 540-742, Korea

**Keywords:** Osteoclast differentiation, Matairesinol, MAP kinases, NFATc1

## Abstract

**Background:**

Matairesinol is a plant lignan present in a wide variety of foodstuffs such as seeds, vegetables and fruits. It has various biological functions including anti-angiogenic, anti-cancer and anti-fungal activities, but its anti-osteoporotic activity, if any, is unknown.

**Methods:**

For osteoclast differentiation, primary mouse bone marrow-derived macrophage cells (BMMs) were cultured for 4 days in the presence of RANKL and M-CSF with the vehicle (DMSO) or matairesinol. Cell cytotoxicity was examined by CCK-8 assay. Gene expression of NFATc1, TRAP, OSCAR, v-ATPasev0d2 were observed in the presence or absence of matairesinol (10 μM) for the indicated times. For evaluating the involvement of NFATc1 in the anti-osteoclastogenic action of matairesinol, BMMs were infected with pMX-IRES-GFP or pMX-IRES-CA-NFATc1-GFP for 8 h with polybrene, and then infected BMMs were cultured with M-CSF and RANKL for 4 days in the presence or absence of matairesinol (10 μM). MAPK signaling activation was examined by immunoblotting. For measuring the resorptive activity of mature osteoclasts, osteoclasts and osteoblasts were co-cultured on BioCoat Osteologic MultiTest slides, and treated with matairesinol for 24 h.

**Result:**

Here we show that matairesinol dose-dependently inhibited the RANKL-induced differentiation of BMMs into osteoclasts by downregulating RANKL-induced expression and activity of NFATc1. Ectopic overexpression of NFATc1 blunted the anti-osteoclastogenic effect of matairesinol implicating NFATc1 in the action of matairesinol. Additionally, matairesinol blocked the RANKL-induced activation of p38 and ERK in BMMs, but had no effect on bone resorption activity in mature osteoclasts.

**Conclusion:**

Taken together, our results suggest that the anti-osteoporotic activity of matairesinol could arise from its anti-osteoclastogenic potential via p38/ERK-NFATc1 signaling, but not by way of anti-resorptive action.

## Background

Bone is a highly dynamic tissue continuously remodeled by osteoclasts and osteoblasts, which are responsible for bone resorption and bone formation, respectively [1]. The delicate balance between osteoclast-mediated bone destruction and osteoblast-mediated bone formation is important for maintaining bone mineral density.

Multinucleated osteoclasts are formed and functionalized by the fusion of macrophage precursor cells. Specifically, excessive bone resorption by overactivated osteoclasts is involved in several lytic bone diseases, such as osteoporosis, periodontal disease and rheumatoid arthritis [2,3].

Osteoporosis is a metabolic disease characterized by decreased bone mass and an increased risk of skeletal fracture and is widely recognized as a major public health problem in an aging society [4]. Several anti-resorptive agents such as bisphosphonates, calcitonin and estrogen have been developed to treat osteoporosis, but each one has side effects including induction of breast cancer, osteonecrosis and vaginal bleeding [5,6]. Thus, a much safer therapeutic strategy for preventing and/or treating lytic bone diseases including osteoporosis is required.

**Figure 1:**
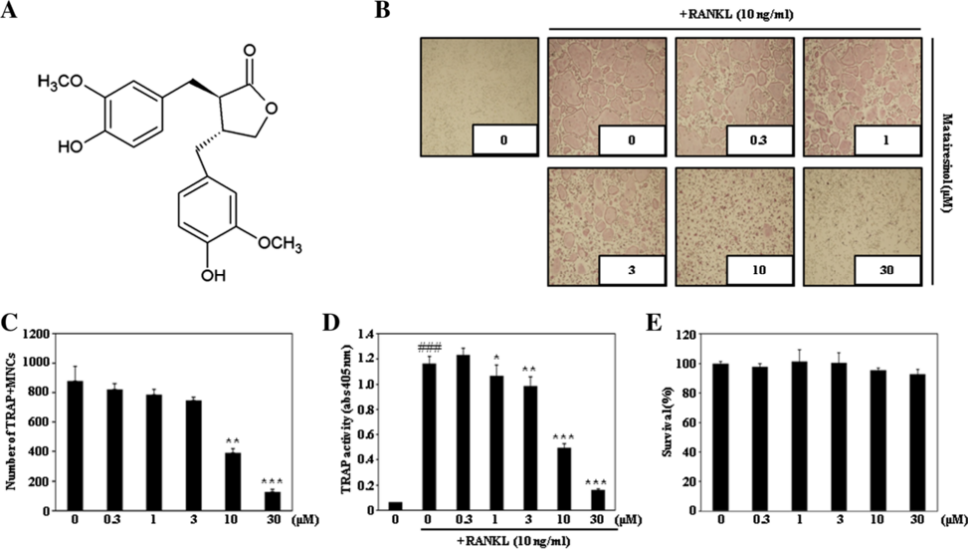
**Matairesinol inhibits RANKL-mediated osteoclast differentiation. (A)** Structure of matairesinol. **(B)** BMMs were cultured for 4 days in the presence of RANKL (10 ng/ml) and M-CSF (30 ng/ml) with the vehicle (DMSO) or matairesinol. Multinucleated osteoclasts were visualized by TRAP staining. **(C)** TRAP-positive multinuclear cells (MNCs) were counted. **, *P* < 0.01; ***, *P* < 0.001 (versus the control). **(D)** TRAP activity was measured. ^###^, *P* < 0.001 (versus the negative control); *, *P* < 0.05; **, *P* < 0.01; ***, *P* < 0.001 (versus the RANKL-treated control). **(E)** Effect of matairesinol on the viability of BMMs was evaluated using the CCK-8 assay.

Natural product-derived small molecules have been used as therapeutic agents for preventing and curing a number of diseases [7,8]. Among them, lignans are phytochemicals elaborated from two phenylpropanoid units in plants and are present in a wide variety of plant foodstuffs including seeds, vegetables, and fruits [9,10]. Matairesinol (Figure 1A), a dibenzylbutyrolactone lignan, has been reported to possess anti-oxidative, estrogenic, or anti-estrogenic activities and reduce the risk of hormone-dependent cancer [11]. However, the detailed anti-osteoporotic activity and mechanism of matairesinol has not been explored. Therefore, we examined the *in vitro* effect of matairesinol on the receptor activator of nuclear factor-κB ligand (RANKL)-induced osteoclast differentiation and the bone resorptive activity of mature osteoclasts.

## Methods

### Reagents and antibodies

Penicillin, streptomycin, cell culture medium, and fetal bovine serum (FBS) were purchased from Invitrogen Life Technologies. Mouse soluble macrophage-colony stimulating factor (M-CSF) and RANKL were purchased from R&D Systems. The CCK-8 assay kit was purchased from Dojindo Molecular Technologies. Antibodies against nuclear factor of activated T cells (NFAT)c1, c-Fos, and actin were purchased from Santa Cruz Biotechnology and antibodies against MAP kinases from Cell Signaling Technology. Matairesinol was purchased from Sigma-Aldrich and dissolved in DMSO (dimethylsulfoxide; Sigma-Aldrich).

### Preparation of osteoclast precursor cells

All experiments were carried out as described in a previous study, with modifications [12]. All animal procedures were performed according to the guide for the Institutional Animal Care and Use Committee of the Korea Research Institute of Chemical Technology (Protocol ID No. 7D-M1). Five-week-old male ICR (Damul Science Co. Deajeon, Korea) were maintained in a room illuminated daily from 07:00 to 19:00 (12:12 h light/dark cycle), with controlled temperature (23 ± 1°C) and ventilation (10–12 times per hour), and humidity was maintained at 55 ± 5% with free access to a standard animal diet and tap water. Bone marrow cells were obtained from five-week-old male ICR mice by flushing femurs and tibias with α-MEM-containing antibiotics (100 units/ml penicillin, 100 μg/ml streptomycin). Bone marrow cells were cultured on culture dishes for 1 day in α-MEM containing 10% FBS and M-CSF (10 ng/ml). Non-adherent bone marrow cells were plated on Petri dishes and cultured for 3 days in the presence of M-CSF (30 ng/ml). After non-adherent cells were washed out, adherent cells were used as bone marrow-derived macrophages (BMMs).

### Osteoclast cell culture and osteoclast differentiation

BMMs were maintained in α-MEM supplemented with 10% FBS, 100 units/ml penicillin, and 100 μg/ml streptomycin. The medium was changed every 3 days in a humidified atmosphere of 5% CO_2_ at 37°C. To differentiate osteoclasts from BMMs, BMMs (1 × 10^4^ cells/well in a 96-well plate) were cultured with M-CSF (30 ng/ml) and RANKL (10 ng/ml). After 3 to 4 days, multinucleated osteoclasts were observed.

### Cell viability assay

BMMs were plated in a 96-well plate at a density of 1 × 10^4^ cells/well in triplicate. After being treated with M-CSF (30 ng/ml) and matairesinol, cells were incubated for 3 days, and cell viability was measured using CCK-8 according to the manufacturer’s protocol.

### TRAP staining and activity assay

Mature osteoclasts were visualized by staining tartrate-resistant acid phosphatase (TRAP), a biomarker of osteoclast differentiation. Briefly, multinucleated osteoclasts were fixed with 3.7% formalin for 10 min, permeabilized with 0.1% Triton X-100 for 10 min, and stained with TRAP solution (Sigma-Aldrich). TRAP-positive multinucleated osteoclasts (MNC; nuclear ≥3) were counted. To measure TRAP activity, multinucleated osteoclasts were fixed in 3.7% formalin for 5 min, permeabilized with 0.1% Triton X-100 for 10 min, and treated with TRAP buffer (100 mM sodium citrate, pH 5.0, 50 mM sodium tartrate) containing 3 mM *p*-nitrophenyl phosphate (Sigma-Aldrich) at 37°C for 5 min. Reaction mixtures in the wells were transferred to new plates containing an equal volume of 0.1 N NaOH, and optical density values were determined at 405 nm.

### RNA isolation and RT-PCR

Total RNA was isolated with TRIzol reagent (Invitrogen) according to the manufacturer’s recommended protocol. Reverse transcription was performed with 1 μg of RNA using oligo(dT) primers, dNTP, buffer, DTT, RNase inhibitor, and SuperScript II reverse transcriptase (Invitrogen). The cDNA was amplified using a TOPsimple DryMIX premix PCR kit (Enzynomics). Table 1 lists the primer sets used in this study. PCR products were electrophoresed on a 1% agarose gel stained with ethidium bromide.

**Table 1:** Primer sequences used in this study

**Target gene**	**Forward (5′–3′)**	**Reverse (5′–3′)**
NFATc1	GGGTCAGTGTGACCGAAGAT	GGAAGTCAGAAGTGGGTGGA
TRAP	ACTTCCCCAGCCCTTACTAC	TCAGCACATAGCCCACACCG
OSCAR	GAACACCAGAGGCTATGACTGTTC	CCGTGGAGCTGAGGAAAAGGTTG
v-ATPasev0d2	ATGGGGCCTTGCAAAAGAAA	GCTAACAACCGCAACCCCTC
GAPDH	ACCACAGTCCATGCCATCAC	TCCACCACCCTGTTGCTGTA

### Western blot analysis

Cultured cells were washed with ice-cold phosphate-buffered saline (PBS) and lysed in lysis buffer (50 mM Tris–HCl, 150 mM NaCl, 5 mM EDTA, 1% Triton X-100, 1 mM sodium fluoride, 1 mM sodium vanadate, and 1% deoxycholate) containing protease inhibitors. Lysates were boiled in sodium dodecyl sulfate (SDS) sample buffer for 5 min, subjected to 10% or 12% SDS-polyacrylamide gel electrophoresis, and transferred to a polyvinylidene difluoride (PVDF) membrane (Millipore). Then, the transferred PVDF membrane was then washed with TBST (10 mM Tris–HCl, pH 7.5, 150 mM NaCl, 0.1% Tween 20) and incubated in the blocking TBST, with 5% skim milk. The membrane was probed with the indicated primary antibody, washed three times for 30 min, incubated with secondary antibody conjugated to horseradish peroxidase for 2 h, and washed three times for 30 min. Membranes were developed with SuperSignal West Femto Maximum Sensitivity Substrate (Pierce) using the LAS-3000 luminescent image analyzer (Fuji Photo Film Co., Ltd., Japan).

### Retrovirus preparation and infection

Retrovirus packaging was described previously [13]. In brief, to isolate the retrovirus, pMX-IRES-GFP (the control retrovirus vector encoding GFP, green fluorescent protein) and pMX containing constitutively active (CA)-NFATc1 were transiently transfected into Plat-E cells (platinum-E retrovirus packaging cell line; Cell Biolabs, Inc.) with Lipofectamine 2000 (Invitrogen, USA) according to the manufacturer’s protocol. Viral supernatant was collected from the culture media 48 h after transfection. BMMs were incubated with viral supernatant in the presence of polybrene (10 μg/ml) for 8 h. The infection efficiency of the retrovirus was determined by GFP expression and was always greater than 80%. After infection, BMMs were induced to differentiate in the presence of M-CSF (30 ng/ml) and RANKL (10 ng/ml) for 4 days.

### Bone pit formation analysis

Mature osteoclasts were prepared by isolating osteoblasts from the calvariae of newborn mice by serial digestion in collagenase (Gibco, Paisley, UK), as previously described [14]. Bone marrow cells were isolated as described above. Osteoblasts and bone marrow cells were co-cultured on a collagen-coated 90-mm dish in the presence of 1α, 25-dihydroxyvitamin D_3_, prostaglandin E_2_ for 6 days. Co-cultured cells were detached from the collagen-coated dishes, re-plated on BioCoat Osteologic MultiTest slides in a 96-well plate, and treated with matairesinol for 24 h. Cells on these slides were stained for TRAP and photographed under a light microscope at 40× magnification. For observation of resorption pits, the slides were washed with PBS and treated with 5% sodium hypochlorite for 5 min. After the plate was washed with PBS buffer and dried it, it was photographed under a light microscope. Quantification of resorbed areas was performed using the ImageJ program.

### Statistical analysis

All quantitative values are presented as mean ± standard deviation. Each experiment in triplicate was performed three to five times, and the figures show results from one representative experiment. Statistical differences were analyzed using the Student’s *t-*test, and a value of *p* < 0.05 was considered significant.

## Results

### Matairesinol inhibits RANKL-induced osteoclast differentiation

To determine the effect of matairesinol on RANKL-induced osteoclast differentiation, BMMs were incubated with matairesinol followed by RANKL treatment. RANKL induced numerous TRAP-positive multinucleated osteoclasts from BMM, but matairesinol inhibited the formation of TRAP-positive multinucleated cells in a dose-dependent manner (Figure 1B, C). Matairesinol also significantly decreased TRAP activity (Figure 1D). To exclude the possibility that the inhibitory effect of matairesinol on osteoclast differentiation might arise from its cytotoxicity *per se*, its effect on the survival of BMMs was further evaluated. As shown in Figure 1E, matairesinol did not exhibit any cytotoxicity at the concentrations used in this study.

### Matairesinol inhibits RANKL-induced expression of NFATc1

**Figure 2:**
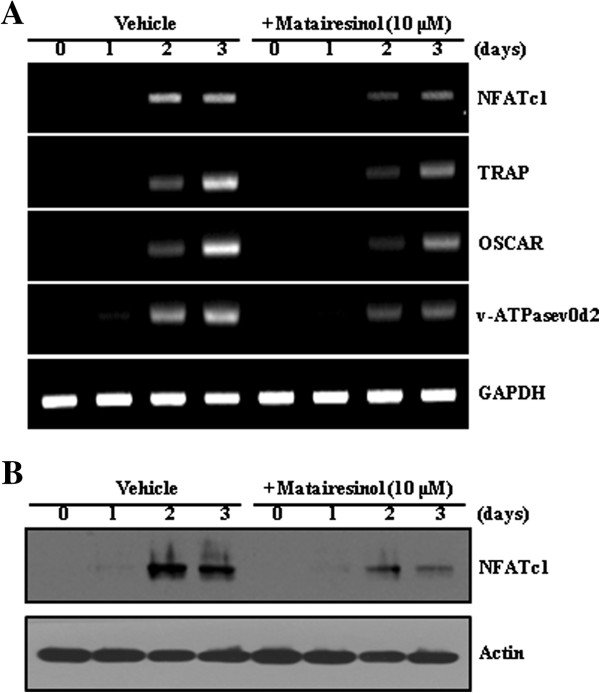
**Matairesinol inhibits RANKL-induced expression of NFATc1. (A)** BMMs were stimulated with RANKL (10 ng/ml) and M-CSF (30 ng/ml) in the presence or absence of matairesinol (10 μM) for the indicated times. Total RNA was then isolated using TRIzol reagent, and mRNA expression levels were evaluated by RT-PCR. Glyceraldehyde-3-phosphate dehydrogenase (GAPDH) was used as the internal control. **(B)** Effect of matairesinol on protein expression level of NFATc1 was evaluated by Western blot analysis. Actin was used as the internal control.

The inhibitory effect of matairesinol on osteoclast differentiation was confirmed by evaluation of expression of several osteoclastogenesis-associated genes including transcriptional factors required for osteoclast differentiation. As shown in Figure 2A, RANKL strongly induced the mRNA expression of NFATc1, but matairesinol attenuated its induction. Matairesinol also strongly attenuated the mRNA expression of NFATc1-dependent genes such as TRAP, osteoclast-associated immunoglobulin-like receptor (OSCAR), and the d2 isoform of vacuolar ATPase V_0_ domain (Atp6v0d2). Western blot analysis further revealed that matairesinol exposure resulted in decreased RANKL-mediated induction of NFATc1 protein (Figure 2B). Taken together, these results suggested that the inhibitory effect of matairesinol on osteoclast differentiation could arise from its potential to inhibit the expression of NFATc1, the key major transcription factor required for osteoclast differentiation.

### Overexpression of NFATc1 restores matairesinol-mediated inhibition of osteoclast differentiation

**Figure 3:**
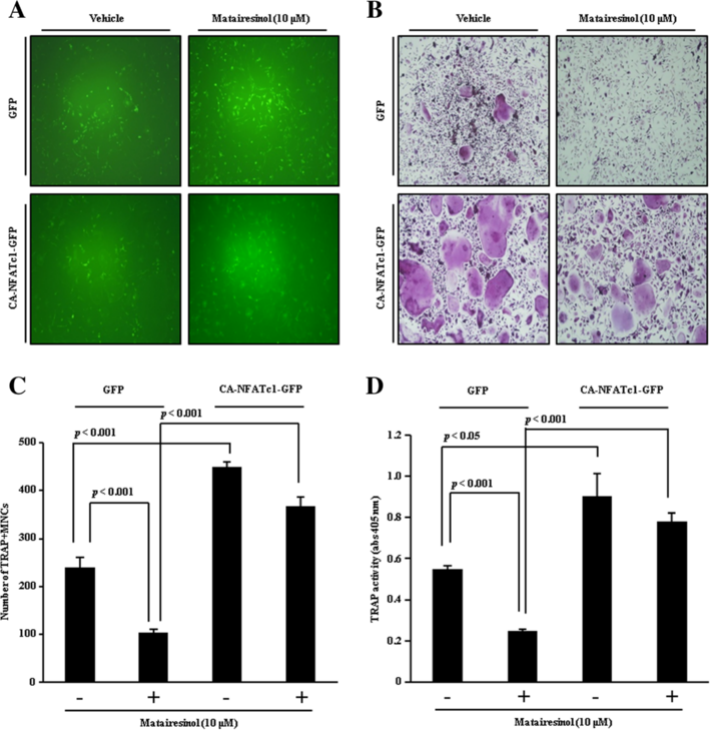
**NFATc1 overexpression restores matairesinol-mediated inhibition of osteoclast differentiation. (A)** BMMs were infected with pMX-IRES-GFP (GFP) or pMX-IRES-CA-NFATc1-GFP (CA-NFATc1-GFP) for 8 h with polybrene (10 μg/ml). Infected BMMs were cultured with M-CSF (30 ng/ml) and RANKL (10 ng/ml) for 4 days in the presence or absence of matairesinol (10 μM). After 4 days, cells were fixed and GFP expression visualized under a fluorescence microscope. **(B)** BMMs were infected with GFP or CA-NFATc1-GFP, and cells were cultured as described in **(A)**. After 4 days, mature TRAP-positive multinucleated osteoclasts were visualized by TRAP staining. **(C)** TRAP-positive cells were counted as osteoclasts. **(D)** TRAP activity was measured at 405 nm.

To investigate whether suppression of NFATc1 expression is crucial for the anti-osteoclastogenic activity of matairesinol, the effect of NFATc1 overexpression on matairesinol-mediated inhibition of osteoclast differentiation was evaluated. When BMMs were infected with retrovirus harboring the control GFP or a constitutively active (CA)-NFATc1-GFP gene expression construct, the infection yield did not differ between BMMs with the control GFP and those with CA-NFATc1-GFP (Figure 3A). Consistent with the result shown in Figure 1B, the TRAP-positive multinucleated osteoclasts were less formed in the presence of matairesinol (upper images in Figure 3B), but the forced expression of NFATc1 dramatically overcame the anti-osteoclastogenic action of matairesinol (bottom images in Figure 3B). The restoring effect of NFATc1 overexpression on the matairesinol-induced inhibition of osteoclast differentiation was also confirmed by counting the number of multinucleated osteoclasts and measuring the activity of TRAP (Figure 3C, D). Taken together, these results confirmed our hypothesis that matairesinol could inhibit RANKL-induced osteoclast differentiation by suppressing the expression of NFATc1.

### Matairesinol inhibits RANKL-induced phosphorylation of p38 and ERK

**Figure 4:**
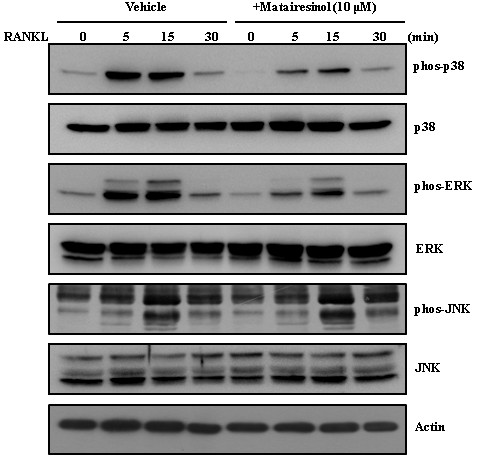
**Matairesinol inhibits RANKL-induced phosphorylation of p38 and ERK.** In the condition of serum starvation for 2 h, BMMs were pretreated with vehicle or matairesinol (10 μM) for 1 h prior to RANKL stimulation (10 ng/ml) at the indicated time periods. Protein expression levels were then evaluated by Western blot analysis. Actin was used as the internal control.

To elucidate the mode of anti-osteoclastogenic action of matairesinol, we investigated whether matairesinol can affect the activation of RANKL-induced early signaling pathways. As shown in Figure 4, RANKL strongly induced the phosphorylation of p38 and ERK, but matairesinol dramatically inhibited those inductions. Our results suggest that inhibition of p38 and ERK phosphorylation could contribute to the anti-osteoclastogenic action of matairesinol.

### Matairesinol has no effect on survival and resorptive activity of mature osteoclasts

**Figure 5:**
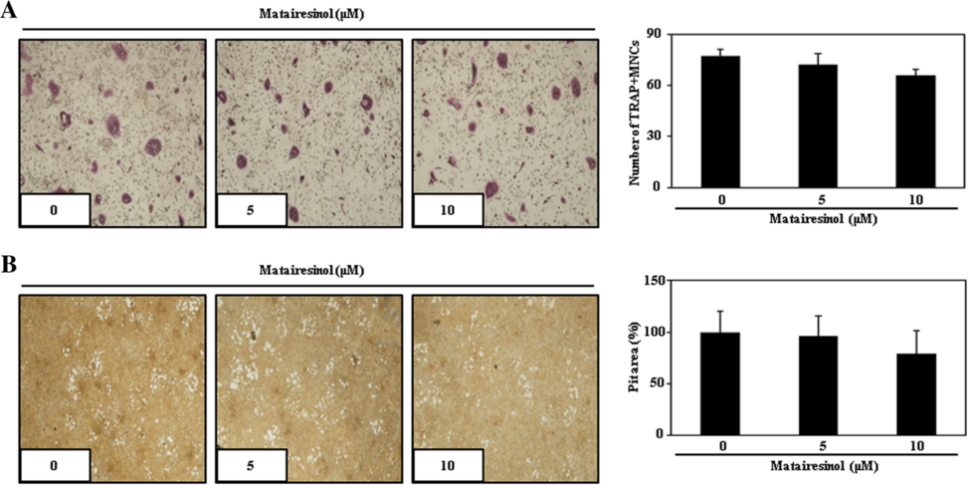
**Matairesinol did not affect survival or anti-resorptive activity of mature osteoclasts. (A)** Mature osteoclasts were plated on BioCoat slides and treated for 24 h with the indicated concentrations of matairesinol. Cells were fixed, permeabilized, stained with TRAP, and photographed under a light microscope. TRAP-positive multinucleated cells were counted. **(B)** Attached cells on BioCoat Osteologic MultiTest slides were removed and photographed under a light microscope. Pit areas were quantified using the ImageJ program.

To investigate whether matairesinol can affect the survival and resorptive activity of mature osteoclasts, we performed both cell counting and the pit formation assay with purified mature osteoclasts in the presence or absence of matairesinol. When purified mature osteoclasts from co-culture were re-plated on carbonate apatite-coated plates and cultured in the presence or absence of matairesinol for 1 day, matairesinol did not show any cytotoxicity and did not affect the number of TRAP-positive multinucleated cells (Figure 5A). Furthermore, the addition of matairesinol did not significantly change the resorptive activity of mature osteoclasts; matairesinol-treated osteoclasts resorbed the carbonate apatite-coated plates similarly to untreated control osteoclasts (Figure 5B). These results indicated that matairesinol did not affect the survival and bone-resorptive activity of mature osteoclasts.

## Discussion

Osteoclasts are present only in bone, where they play an essential role in bone resorption. Intervention in osteoclast differentiation and its function has been postulated as a treatment for bone metabolic diseases such as osteoporosis [15].

RANKL signaling triggers osteoclast differentiation and has been an important target for treating pathological bone loss. Docking of RANKL to its receptor, RANK, rapidly activates MAP kinases such as p38, ERK, and JNK. These MAP kinases are essential for the differentiation, survival and activation of osteoclasts [16-18]. Activated MAP kinases then lead to the stimulation of transcription factors such as NFATc1.

NFATc1 is a master regulator of osteoclast differentiation. The expression of NFATc1 is strongly up-regulated during RANKL-induced osteoclastogenesis. The overexpression of NFATc1 accelerates RANKL-induced osteoclast differentiation, and transduced-NFATc1 increased osteoclast formation even without stimulation with RANKL; in contrast, NFATc1-deficent embryonic stem cells fail to differentiate into osteoclasts even in the presence of RANKL [19]. In this study, we found that matairesinol strongly attenuated RANKL-induced osteoclast differentiation with suppression of NFATc1 expression. The inhibitory effect of matairesinol via downregulation of NFATc1 was confirmed by evaluating the mRNA expression levels of NFATc1-dependent genes such as TRAP, OSCAR, and v-ATPasev0d2. NFATc1 induces the expression of TRAP and OSCAR during osteoclast differentiation [20] and plays a role in the process of osteoclast multinucleation through induction of Atp6v0d2 [21]. The results of the NFATc1 overexpression experiment also confirmed the involvement of NFATc1 in the anti-osteoclastogenic action of matairesinol. The matairesinol-mediated inhibition of osteoclast differentiation was almost rescued by the ectopic expression of NFATc1.

As mentioned above, during osteoclastogenesis, MAP kinases regulate NFATc1. In particular, Huang et al. [22] reported the RANKL-dependent role of p38 in modulating NFATc1 expression during osteoclastogenesis. Furthermore, the involvement of the ERK pathway in osteoclast differentiation has received much attention; ERK can trigger activation of c-Fos-NFATc1 for osteoclastogenesis, and the inhibition of ERK suppresses osteoclast formation and function [23]. Likewise, pharmacologic inhibition studies have suggested the importance of p38 and ERK signaling in osteoclastogenesis [24]. Therefore, modulation of p38 and ERK could control RANKL-mediated osteoclast differentiation via NFATc1-dependent transcription. In this study, matairesinol suppressed RANKL-induced activation of p38 and ERK. Taken together with the inhibitory activity of matairesinol in downregulating NFATc1, its anti-osteoclastogenic activity could result from its potential to inhibit RANKL-induced activation of p38 and ERK signaling.

Of importance, numerous drugs targeting the resorptive action of osteoclasts including bisphosphonate, cathepsin K inhibitors, and src inhibitors, are currently available or under clinical examination to treat bone metabolic diseases [25], and several phytochemicals with anti-osteoclastogenic activity also inhibit the survival and/or the resorptive activity of mature osteoclasts [26,27]. For this reason, we further evaluated the anti-resorptive activity of matairesinol, but found that it did not affect survival and bone resorptive activity of mature osteoclasts. These results suggested that matairesinol with anti-osteoclastogenic activity via the down-regulation of the p38/ERK-NFATc1 signaling axis might not be enough to inhibit the survival and function of mature osteoclasts.

## Conclusions

To our knowledge, this study is the first to report the anti-osteoclastogenic activity of matairesinol: It is associated with downregulation of p38, ERK, and NFATc1, which consequently leads to the decreased expression of genes required for osteoclastogenesis such as TRAP, OSCAR, and v-ATPasev0d2. Although matairesinol did not inhibit the survival and bone resorptive activity of mature osteoclasts, it could be useful for preventing osteoclastogenesis-related bone metabolic diseases including osteoporosis, rheumatoid arthritis and periodontal disease.

## Competing interests

The authors declare that they have no competing interests.

## Authors’ contributions

SWC carried out osteoclast differentiation, cell cytotoxic assay, RT-PCR, western blotting, retrovirus infection and drafted the manuscript. KIP supervised of data interpretation and manuscript preparation. JTY carried out bone resorptive activity. BJR carried out data analysis. KJK carried out data analysis and draft the manuscript. SHK supervised the study design, data interpretation and corrected the manuscript for publication. All authors read and approved the final manuscript.

## Pre-publication history

The pre-publication history for this paper can be accessed here:

http://www.biomedcentral.com/1472-6882/14/35/prepub
